# *Microbacterium spp*. peritonitis in patients undergoing peritoneal dialysis: a single-center experience and literature review

**DOI:** 10.3389/fmed.2023.1297296

**Published:** 2023-11-23

**Authors:** Xiao Yu, Zhao Li, Xinbo Yang, Xiaopei Wang, Di Nan, Wanhong Lu, Jing Lv, Ying Ma

**Affiliations:** ^1^Department of Nephrology, Kidney Hospital, The First Affiliated Hospital of Xi'an Jiaotong University, Xi'an, Shaanxi, China; ^2^Department of Nephrology, The Second Affiliated Hospital of Shaanxi University of Traditional Chinese Medicine, Xianyang, China

**Keywords:** *Microbacterium spp*., peritoneal dialysis (PD), peritonitis, vancomycin, meropenem

## Abstract

**Introduction:**

Peritoneal dialysis-related peritonitis (PDRP) caused by *Microbacterium spp*. is very rare, with only 9 cases reported to date. In this study, we report the treatment experiences of 7 patients at our peritoneal dialysis center.

**Methods:**

We retrospectively collected clinical characteristics and antibiotic management of all 7 episodes of PDRP caused by *Microbacterium spp*. in 7 patients from at our center over 4 years, and reviewed the documented *Microbacterium spp*. PDRP in the literature.

**Results:**

Empiric antibiotic therapy was initiated as soon as possible, and consisted of intraperitoneal (IP) gentamicin in combination with vancomycin. After up to 5 days, gentamicin was changed to meropenem if the treatment was not effective. The intended course of antibiotic treatment was 21-day. Totally, 6 episodes were cured (85.7%), which was higher than reported.

**Conclusion:**

The 21-day antibiotic therapy program by combining vancomycin and meropenem may benefit the management of *Microbacterium spp*. PDRP.

## Introduction

Peritoneal dialysis (PD)-related peritonitis (PDRP) is a significant complication in patients undergoing PD, leading to hospitalization, catheter loss, technique failure, conversion to hemodialysis, and death (1). *Microbacterium spp*. is a genus of aerobic Gram-positive bacteria present in the environment, characterized by rod-shaped Gram-positive bacilli that are non-sporulating, acid-resistant, aerobic, and weakly anaerobic, primarily undergoing respiratory metabolism with occasional weak fermentation. Their nutritional requirements are complex. PDRP caused by *Microbacterium spp*. is rare, with only nine cases reported to date. In this study, we summarized the treatment experiences of the seven *Microbacterium* species episodes at our center and reviewed previously reported cases, trying to explore potential approaches that may benefit the cure rate of *Microbacterium spp*. peritonitis.

## Materials and methods

We reviewed the records of PDRP cases identified as *Microbacterium spp*. infections at the center of the First Affiliated Hospital of Xi'an Jiaotong University from January 2019 to April 2023. Clinical and demographic data included age, gender, and ESRD cause. For the *Microbacterium spp*. peritonitis episodes, we also collected precise data on *Microbacterium* species and potential risk factors, such as catheter-related infection, body mass index (BMI), and personal exposure including keeping domestic pets, weeding or growing crops, and Charlson Comorbidity Index [CCI, the CCI index assigns 1 point for the history of myocardial infarction, congestive heart failure, peripheral vascular disease, cerebrovascular disease (transient ischemic attack or cerebrovascular accident with minor or no residua), dementia, chronic pulmonary disease, connective tissue disorder, peptic ulcer disease, mild liver disease, and diabetes without end-organ damage; 2 points for hemiplegia, moderate-to-severe renal disease, diabetes with end-organ damage, tumor without metastases, leukemia, lymphoma, and myeloma; 3 points for moderate-to-severe liver disease; and 6 points for metastatic solid tumor or acquired immunodeficiency syndrome. For every decade over 40 years of age, 1 point is added to the score] (1). Additionally, PD duration, residual glomerular filtration rate (rGFR), catheter-related infections, clinical features of peritonitis, and both blood and dialysate examination results, including blood cell counts, serum albumin, serum potassium, C-reactive protein (CRP), procalcitonin (PCT), effluent white blood cell count, and microbiologic causes, were retrospectively collected.

**Figure 1:**
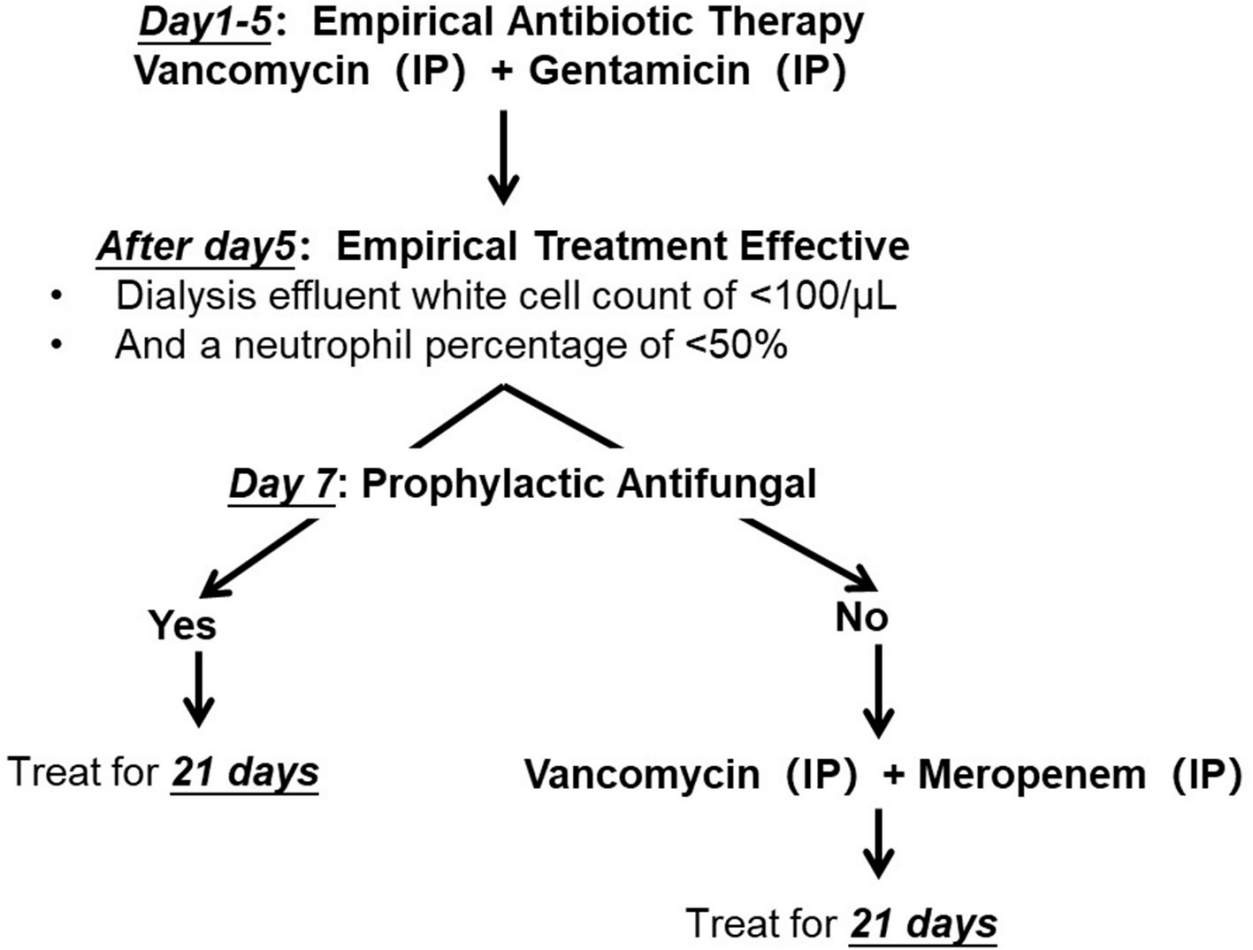
Antibiotic protocol of *Microbacterium spp*. peritoneal dialysis related peritonitis. IP, introperitoneal administration.

Peritonitis was diagnosed according to the peritonitis definition in the 2022 International Society for Peritoneal Dialysis (ISPD) guidelines. *Microbacterium spp*. peritonitis was enrolled based on dialysate culture. To increase the yield of peritoneal effluent culture, we directly inoculated the effluent into rapid blood culture bottle kits (aerobic and anaerobic, BacT/Alert, bioMérieux, Inc., Basingstoke, UK). Our center has not provided drug sensitivity results due to limited technical experience. According to the ISPD guidelines, we initiated empiric antibiotic therapy as soon as possible. All patients enrolled in the outpatient service received intermittent IP gentamicin at a dose of 0.6 mg/kg/d, up to a maximum of 40 mg in one PD exchange, in combination with IP vancomycin (administered at a dose of 15 mg/kg of body weight rounded to the nearest 500 mg to the longest dwell bag for at least 6 h). Vancomycin was used in accordance with the vancomycin-monitoring protocol previously reported (2). After 5 days of empiric treatment, antibiotic therapy was adjusted according to the treatment effect. We defined a dialysis effluent white cell count of <100/μl and a neutrophil percentage of <50% as treatment-effective. Gentamicin was changed to meropenem (1 g/day by IP injection) if the empirical treatment was not effective. Prophylactic antifungal therapy (fluconazole, 0.2 g/day) was administered 7 days after combination antibiotic treatment (Figure 1).

We performed a literature search on PubMed, Wiley, Nature, the National Center for Biotechnology Information, and the China National Knowledge Infrastructure using the search terms “*Microbacterium*” and “peritoneal dialysis-related peritonitis.” We collected the general information of the reported patients infected by *Microbacterium spp.*, such as sex, age at onset, and clinical features. In addition, we summarized the characteristics of all patients, clinical history, characteristics of antibiotic sensitivities, antibiotic treatment regimen, duration of treatment, and treatment outcome according to the ISPD guideline recommendation (3). Additionally, the exit site is cleansed using sterile saline solution every day or every 2 days, and mupirocin cream is applied to the catheter exit site and an exit site dressing cover is used to prevent catheter-related infection (4).

Follow-up was conducted until 31 July 2023, and outcomes for all episodes were identified and classified as medical cure, refractory, recurrence, and relapsing as recommended by the ISPD guideline (3). In refractory peritonitis cases, catheter removal is indicated to preserve the peritoneum for future PD and prevent morbidity and mortality. However, we propose that if the PD effluent white cell count is decreasing toward normal, it may be appropriate to observe the antibiotic effect for longer than 5 days instead of mandating PD catheter removal by day 5. The final decision on whether to remove the catheters depends on the clinical severity and the options available to the patients. In cases where the patient's clinical condition is not deteriorating and they prefer to continue with PD, we aim to minimize premature or unnecessary PD catheter removal.

## Results

According to the ISPD recommendations, seven patients were diagnosed with PDRP (3), and Table 1 shows their demographic and clinical characteristics. The average age was 47.4 ± 18.7 years, and all were men. Chronic glomerulonephritis is the leading cause of end-stage renal disease. Their median PD duration was 51 months. All the patients in our study were on continuous ambulatory peritoneal dialysis (CAPD) using a glucose-based acidic lactate PD solution. Total KT/V urea was 1.97 ± 0.52/week. The common manifestations of the peritonitis were abdominal pain and cloudy dialysis effluent. There was no concomitant catheter-related infection. Because all patients were on dialysis, the minimum CCI is 2. The CCI in Case 3 is as high as 12.

**Table 1:** Characteristics of six patients with *Microbacterium spp*. peritoneal dialysis-related peritonitis at our center.

**Case**	**1**	**2**	**3**	**4**	**5**	**6**	**7**
Gender	Male	Male	Male	Male	Male	Male	Male
Age	28	65	65	29	71	35	39
BMI (kg/m^2^)	27.7	18.9	17	26	23.4	22.5	26.7
Primary disease	HTN	DM	DM	HTN	CGN	CGN	CGN
WBC ( × 10^9^/L)	8.8	6.68	2.85	13.82	9.2	4.22	8.47
NEU (%)	75.1	77.5	79.8	82.4	79.5	72.2	86.2
CRP (mg/L)	18.9	200.5	9.31	8.23	43.8	23.4	48.3
PCT (ng/mL)	0.168	6.76	2.13	0.388	0.401	0.406	0.205
HGB (g/L)	103	101	116	106	138	105	94
ALB (g/L)	29.7	20.3	29.7	36.7	27.8	31.8	28.1
BUN (mmol/L)	15.85	14.75	19.14	20.55	20.54	20.81	17.61
CRE (umol/L)	654	951	491	826	784	538	901
rGFR (ml/min/1.73m^2^)	8	6	7	6	6	13	5.7
Chol (mmol/L)	5.06	3.29	4.2	4.54	3.74	3.0	3.26
TG (mmol/L)	1.45	1.27	1.11	2.06	1.7	0.54	3.22
Serum potassium (mmol/L)	4.06	3.14	3.29	3.78	3.35	3.75	3.22
Serum calcium (mmol/L)	2.26	1.86	1.89	2.42	2.3	2.15	2.04
Serum phosphorus (mmol/L)	1.79	1.19	1.44	1.9	1.49	1.25	1.29
CCI	2	8	12	2	5	2	2
PD duration (months)	19	180	61	24	51	17	60
tKT/V urea	2.96	N/A	1.59	2.14	1.66	1.69	1.8
PD exchange volume (L/day)	8	9^a^	8	8	8	10	10
PD ultrafiltration volume (ml/day)	450	N/A	430	400	520	670	540
Main symptoms	AP, CPDE	AP, CPDE	AP, CPDE	AP, CPDE	AP, CPDE	AP, CPDE	AP, CPDE
Catheter-related infection (either exit-site or tunnel)	No	No	No	No	No	No	No

^a^This patient received 8 L and 10 L alternately, thus the average PD exchange volume was 9 L/day, with no available PD adequacy test in the last 2 months. HTN, Hypertension; DM, Diabetes mellitus; CGN, Chronic glomerulonephritis; WBC, White blood cell; NEU, Neutrophils; CRP, C-reactive protein; PCT, Procalcitonin; HGB, Hemoglobin; ALB, Albumin; BUN, Urea nitrogen; CRE, Creatinine; CCI, Charlson Comorbidity Index [The CCI index assigns 1 point for history of myocardial infarction, congestive heart failure, peripheral vascular disease, cerebrovascular disease (transient ischemic attack or cerebrovascular accident with minor or no residua), dementia, chronic pulmonary disease, connective tissue disorder, peptic ulcer disease, mild liver disease, and diabetes without end-organ damage; 2 points are assigned for hemiplegia, moderate to severe renal disease, diabetes with end-organ damage, tumor without metastases, leukemia, lymphoma, and myeloma; 3 points are assigned for moderate or severe liver disease; and 6 points are assigned for metastatic solid tumor or acquired immunodeficiency syndrome (AIDS). For every decade over 40 years of age, 1 point is added to the score.]; AP, abdominal pain; CPDE, cloudy PD effluent; F, fever; N/A, not available.

The antibiotic management and clinical outcomes of *Microbacterium spp*. peritonitis at our PD center are presented in Table 2. At our center, we achieved medical cure in six patients (Cases 1, 2, 4, 5, 6, and 7), while one patient (Case 3) experienced relapsing. Throughout the treatment, we continued CAPD. After 3 months of follow-up, Cases 3 and 4 received PD catheter removal due to relapsing and repeat peritonitis, respectively.

**Table 2:** Antibiotic management and clinical outcomes of the *Microbacterium spp*. peritonitis at our PD center.

**Case**	**Species**	**Culture (days)**	**PD effluent routine test (day 0)**		**Empirical antibiotics**	**PD effluent routine test (day 5)**		**Day-5 serum vancomycin level (mg/L)**	**Adjusted antibiotics**	**PD effluent routine test (day 10)**		**PD effluent routine test (day 14)**		**Antibiotic treatment duration (days)**	**Outcome**
			**WBC (**×**10**^6^**/L)**	**PMN (%)**		**WBC (**×**10**^6^**/L)**	**PMN (%)**			**WBC (**×**10**^6^**/L)**	**PMN (%)**	**WBC (**×**10**^6^**/L)**	**PMN (%)**		
1	*M. arborescens*	1.29	942	64.2	IP GM+ VAN	105	65	8.8	IP MEM+VAN	105	25	84	17	21	Medical cure
2	*M. arborescens*	1.08	582	93	IP GM+ VAN	105	39	11.4	IP MEM+VAN	26	39	14	14	21	Medical cure
3	*M. paraoxydans*	1.29	547	94	IP GM+ VAN	315	95	8.9	IP MEM+VAN	62	90	81	21	21	Relapsing (PD catheter removal for cure)
4	*M. arborescens*	1.23	315	93	IP GM+ VAN	278	95	9.5	IP MEM+VAN	36	82	38	29	21	Repeat (PD catheter Removal for cure)
5	*M. arborescens*	3.75	1,454	79	IP GM+ VAN	124	70	16.4	IP MEM+VAN	52	15	11	18	21	Medical cure
6	*M. spp*	2.6	463	65	IP GM+ VAN	681	86	15.1	IP MEM+VAN	44	94	35	3.1	21	Medical cure
7	*M. aurantiacum*	2.12	737	88.6	IP GM+ VAN	259	63.7	10.2	IP MEM+VAN	107	31.8	58	26	21	Medical cure

*M, Microbacterium;* PD, peritoneal dialysis; IP, intraperitoneal; GM, gentamicin; CZ, cefazolin; CAZ, ceftazidime; MSS, mezlocillin sodium/sulbactam; MEM, meropenem; VAN, vancomycin; ONZ, ornidazole. Medical cure, Complete resolution of peritonitis together with NONE of the following complications: relapse/recurrent peritonitis, catheter removal, transfer to hemodialysis for 30 days or death; Refractory, Peritonitis episode with persistently cloudy bags or persistent dialysis effluent leukocyte count >100 × 10^9^/L after 5 days of appropriate antibiotic therapy; Recurrent, Peritonitis episode that occurs within 4 weeks of completion of therapy of a prior episode but with a different organism; Relapsing, Peritonitis episode that occurs within 4 weeks of completion of therapy of a prior episode with the same organism or one sterile (culture negative) episode (i.e., specific organism).

Totally, nine cases of PDRP caused by *Microbacterium spp*. were reported previously, comprising three cases of *M. paraoxydans*, two cases of *M. oxydans*, two cases of *M. arborescens*, one case of *M. resistens*, and one case of *M. aurum*. Table 3 shows general information about the reported patients infected by *Microbacterium spp*. Among the episodes, five episodes (Cases B, C, E, G, and H) experienced medical cure, while four episodes (Cases A, D, F, and I) failed, involving three catheter removals and one refractory peritonitis. Antimicrobial susceptibility testing (AST) revealed 21 antibiotics used in the treatment of these 9 patients.

**Table 3:** Outlines of patients with peritoneal dialysis-related peritonitis identified in the literature.

**Case**	**References**	**Age (years)/sex**	**Duration of PD (years)**	**Species**	**PD effluent routine test**		**Antibiotic treatment initial treatment**	**After drug sensitivity testing**	**Antibiotic treatment (days)**	**Outcome**
					**WBC (**×**10**^6^**/L)**	**PMN (%)**				
A	Wybo et al. (5)	48/Female	1.8	*M. oxydans*	N/A	N/A	IP ATM+VAN (4 days)	Po AM/CA (8 days)	8	Relapsing (day 8)
B	Adams et al. (6)	57/Female	8	*M. arborescens*	N/A	N/A	IP GEN+VAN	IP GEN + VAN (21 days)	21	Repeat (month1), IP GEN+VAN (6 days), until the PD catheter removal.
C	Miyamoto et al. (7)	60/Male	2.3	*M. paraoxydans*	826	74	IP CZ+CAZ (7 days)	IP ERY (14 d), Po SXT (21 days)	21	Medical cure
D	Gallois et al. (8)	71/Male	1.1	*M. resistens*	678	83	IP VAN+CAZ+AMI	IP AMP (7 days) + GEN (3 days)	7	Relapsing (day 6, IP AMP(47 days) + GEN (5 days), until the PD catheter removal)
E	Yusuf et al. (9)	80/Male	NK	*M. aurum*	1070	55	Po CIP+IP VAN (7 days)	Po CIP (21 days)	21	Repeat (month 4), PD catheter removal for cure
F	Yusuf et al. (9)	48/Female	NK	*M. oxydans*	767	64	IPATM+VAN (3 days)	Po AM/CA+ IP VAN (7 days)	10	Refractory and Recurrent (PD catheter removal for cure)
G	Choi et al. (10)	54/Female	1	*M. paraoxydans*	2900	84	IP CZ+CAZ (11 days)	IP VAN, Po CLI (14 days)	14	Medical cure
H	Lam et al. (11)	74/Male	1.4	*M. paraoxydans*	815	78	IP CZ+CAZ (14 days)	No change	14	Repeat (month 2), PD catheter removal for cure.
I	Girişgen et al. (12)	16/Female	3	*M. arborescens*	N/A	N/A	IP CZ+CAZ	IP CPM (14 days)		Relapsing (day 7), PD catheter removal for cure.

N/A, not available; Po, peros; ATM, aztreonam; VAN, vancomycin; AM/CA, amoxicillin/clavulanate; GEN, gentamicin; CZ, cefazolin; CAZ, ceftazidime; CIP, ciprofloxacin SXT, trimethoprim/sulfamethoxazole; ERY, erythromycin; AMP, ampicillin; CLI, clarithromycin; CPM, cefepime; AMI, amikacin.

**Figure 2:**
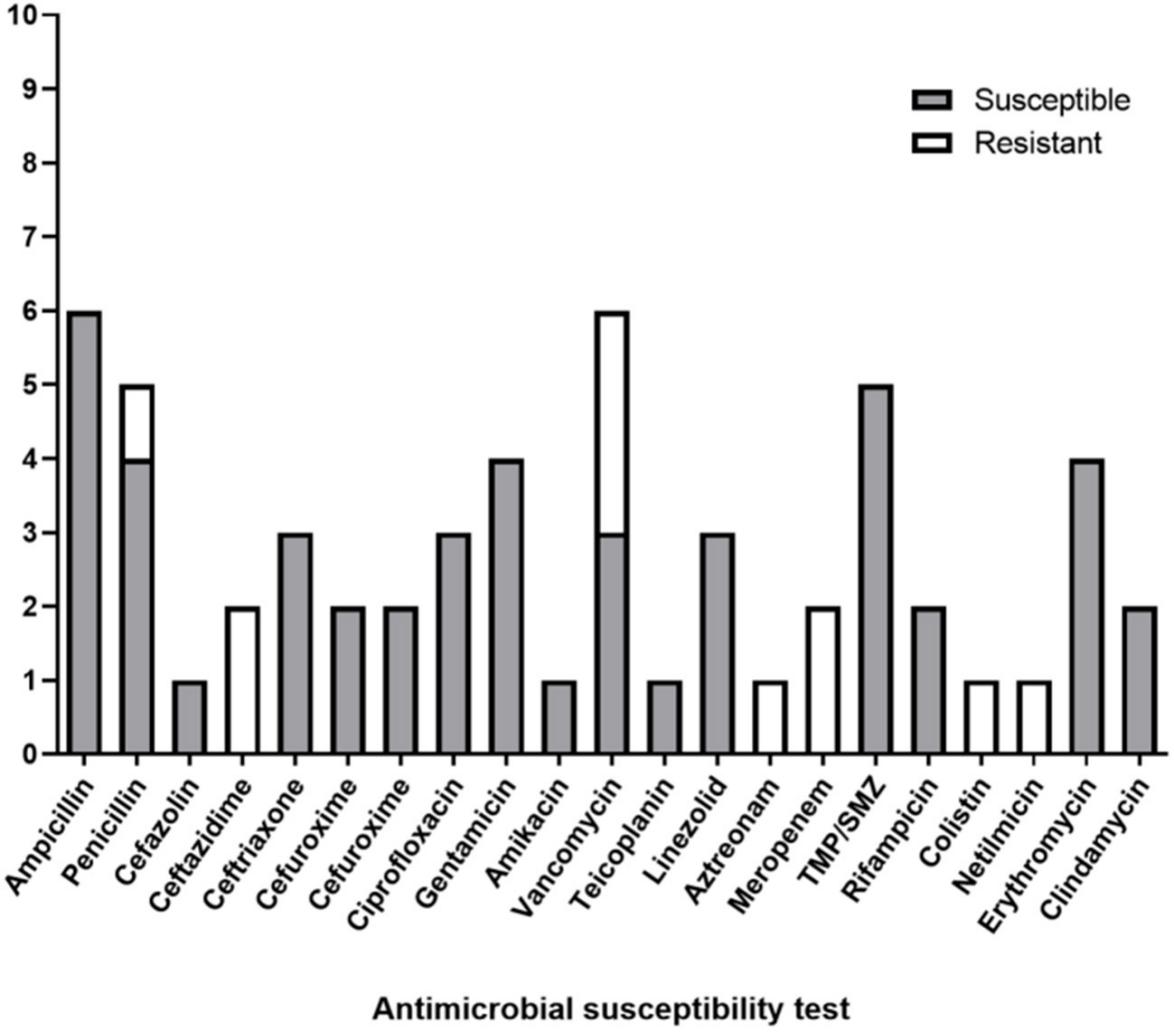
The characteristics of antibiotic sensitivities in literature reviews.

All nine patients underwent AST, and antibiotic therapy was modified until the culture results were known. In the AST, six patients exhibited sensitivity to ampicillin, five patients to trimethoprim/sulfamethoxazole, four patients to penicillin, four patients to gentamicin, four patients to erythromycin, three patients to vancomycin, and three patients to ceftriaxone. However, a few reports mentioned antibiotic resistance, among which three showed resistances to vancomycin, two to ceftazidime, one to cefazolin, one to penicillin, one to aztreonam, one to colistin, and one to netilmicin (Figure 2).

As for the administration of vancomycin in the reported cases, six patients (Cases A, B, D, E, F, and G) received IP vancomycin, and it was employed as an empiric antibiotic in five patients (Cases A, B, D, E, and F); patient A showed susceptibility to vancomycin (minimum inhibitory concentration [MIC] determined by the E-test was 8 mg/L) and improved with IP aztreonam 2 g and vancomycin 1 g in 2 L of dialysate. After 4 days with amoxicillin/clavulanic acid (875 mg twice a day orally), the patient was discharged. However, the patient experienced relapsing on day 8. Patient B was susceptible to vancomycin (MIC determined by the E-test was unknown). Treatment was initiated with IP gentamicin (20 mg/24 h) and vancomycin (2 g/72 h), and vancomycin was continued for 3 weeks while the patient experienced a repeat episode. Patient D was empirically treated with IP vancomycin (12 g/3 d), ceftazidime (1 g/d), and amikacin (2 mg/kg/d). Because of the resistance to vancomycin shown by AST, it was adjusted to IP ampicillin (250 mg, thrice daily) plus IP gentamicin (45 mg/d). After 3 days, gentamicin was discontinued, and the patient was discharged with a prescription of IP ampicillin (250 mg, thrice daily) to be taken for 1 more week. However, the patient experienced a relapse on day 8. Patient E was susceptible to vancomycin. Empirical therapy involved oral ciprofloxacin (MIC 1.0 mg/L) and IP vancomycin. Aerobic culture revealed the growth of two types of organisms (*Acinetobacter spp*. and *Microbacterium aurum*). Oral ciprofloxacin was continued for 3 weeks, but the patient experienced a repeat episode after 4 months. For patient F, empirical therapy with IP vancomycin was initiated, but AST did not contain vancomycin. The aerobic culture grew three types of organisms (*Coagulase-negative staphylococci, Streptococcus mitis*, and *Corynebacterium amycolatum*). He underwent PD catheter removal due to refractory and recurrent peritonitis. Patient G was treated empirically with IP cefazolin (15 mg/kg/day) and ceftazidime (1 g/day) for the first 11 days. On day 12, the antibiotics were changed to IP administration of vancomycin (2 g loading, followed by 1 g every 5 days; the MIC determined by the E-test was 2 μg/ml) and oral administration of clarithromycin (500 mg every 12 h). After 2 weeks of antibiotic administration, the patient was cured.

Three patients were treated without vancomycin, among whom patients C and H were vancomycin-resistant. In detail, the treatment for patient C was changed to intravenous erythromycin (MIC ≤ 0.12 mg/L, 14 days) and oral sulfamethoxazole/trimethoprim (MIC ≤ 0.5 mg/L, 21 days) after AST, and PD was interrupted (hemodialysis with a dual-lumen catheter as vascular access was performed seven times in total). The patient was cured after 3 weeks of antibiotic administration. Patient H received empirical therapy with IP cefazolin (1 gram every 24 h) and ceftazidime (1.5 grams every 24 h) for 2 weeks. However, patient H experienced a repeat episode after 2 months. Furthermore, due to an AST without vancomycin, the treatment of patient I was changed to IP cefepime (CPM, 14 days), and she relapsed after 1 week.

## Discussion

This report was a single-center experience and literature review of PDRP caused by *Microbacterium spp*., comprising seven cases from our center and nine cases from the literature. Clinical features, therapeutic management, and clinical outcomes were collected. In our center, the clinical cure rate was 85.7% (6/7), while in the literature review, it was 55.6% (5/9).

Our six patients (Cases 1, 2, 4, 5, 6, and 7) were cured, and the cure rate was 85.7%, which was higher than reported (55.6%). Importantly, the six cured patients did not experience refractory peritonitis, all-cause hospitalization, technique failure, or death during a follow-up period of 3 months. Only one patient (Case 3) experienced relapsing. In the literature review, although five patients (Cases B, C, E, G, and H) were cured, Cases B and H experienced a repeat episode within 3 months, and patient E experienced a repeat episode after 4 months.

The focus of the literature reviews was on the results of AST and antibiotic management, which revealed that the sensitivity rate of *Microbacterium spp*. to vancomycin was 50%, although the sample size was small. Vancomycin was still used despite treatment failure in some cases, and the serum vancomycin levels were not mentioned. Although the ISPD guideline showed controversy in the relationship between serum vancomycin levels and peritonitis outcomes, we previously discovered that serum vancomycin levels correlate with short-term adverse outcomes of PD-associated peritonitis, and the diagnostic threshold value of day 5 serum vancomycin levels for short-term adverse outcomes was 10.1 mg/L^2^. In this study, the mean serum vancomycin level on day 5 was 11.5 ± 3.1 mg/L in medica-cured cases, which was suboptimal in the relapsing case.

The combination of vancomycin and meropenem as adjusted antibiotic therapy may benefit our cure rate. A review of 50 human specimens (species obtained from blood cultures, wounds, normally sterile anatomical sites, sterile materials, urine, and miscellaneous materials) revealed that *Microbacterium spp*. are susceptible to vancomycin (98% of the isolates were susceptible) (13). This provides the theoretical basis for the continued use of vancomycin to treat *Microbacterium spp*. PDRP. Moreover, meropenem exhibits an ultra-broad spectrum of antibacterial activity, encompassing Gram-positive and Gram-negative aerobes and anaerobes, including numerous strains resistant to other antibacterials (14). Furthermore, in the review of 50 human specimens, all 50 isolates were susceptible to meropenem.

The 21-day antibiotic course may be another potential beneficial measure to improve the cure rate in our center. As for the previously reported cured cases, patients B, C, and E received 21 days of antibiotic treatment, while for patients G and H, the course of antibiotic treatment was 14 days. The 2016 ISPD guideline recommended a 21-day course of effective antibiotic treatment for corynebacterial peritonitis. Considering *Microbacterium spp*. as a genus of coryneform bacteria originally proposed by Orla-Jensen in 1919 (15), we proposed a 21-day treatment duration for our seven patients. Since the 2022 ISPD peritonitis guideline suggested that *Corynebacterium* peritonitis should be treated with effective antibiotics for 2 weeks, a shorter treatment duration in *Microbacterium spp*. peritonitis deserves future observation accordingly.

The high positive rate of *Microbacterium spp*. in our center may be attributed to the improvement of our culture technology. We employed blood culture bottles as the preferred approach for the bacterial culture of PD effluent, which is consistent with the guideline recommendation (3).

Additionally, identifying risk factors associated with *Microbacterium spp*. infection warrants attention. Among our patients, four (four of seven) patients experienced their first peritonitis episode, and all patients had no concomitant catheter-related infections. The average PD duration was 51 months. Meanwhile, given that the incidence of encapsulating peritoneal sclerosis (EPS) increases with the duration of PD, we tried to screen it in our cases. All of our cases lack typical presentations of EPS, such as signs of intestinal obstruction or a high peritoneal transporter status with incipient ultrafiltration failure. In addition, Case 3, whose PD duration was 61 months, underwent a CT scan because of peritonitis relapsing and revealed no evidence of a thickened peritoneal membrane. In terms of occupation, four of seven patients were farmers. Notably, all seven patients were male, which is inconsistent with the literature reports (4/9). It is hard to explain the underlying reasons. Deciphering the above factors may allow for greater progress in prevention and treatment. The CCI was a better predictor than models containing age, diabetes, cardiovascular disease, and albumin and a strong predictor of mortality in incident PD patients. The mortality rate was 50/100 patient-years for patients with a CCI score of 8 or greater (16). In our cases, the CCI of Case 3 was higher than 8 because of cancer and radiotherapy, and he experienced peritonitis relapsing and catheter removal. However, there was no definitive evidence confirming the predictive value of CCI or peritonitis adverse outcomes.

This study had several limitations. It was a single-center experience in treating PDRP caused by *Microbacterium spp*.. The promotion of treatment experience is limited due to the lack of AST. We have communicated with our laboratory in this regard and will promptly enhance the standard operating procedure associated with the AST of *Microbacterium spp*. based on our experiences and the literature reports available.

## Conclusion

The treatment experience of PDRP caused by *Microbacterium spp*. is limited, and the treatment effect in the literature is not satisfactory. In this single-center report, seven cases of *Microbacterium spp*. peritonitis were presented for the first time. Our 21-day antibiotic therapy program based on a combination of IP vancomycin and meropenem achieved a relatively high cure rate. To validate our experience, available AST is needed, and further randomized controlled trials are required.

## Data availability statement

The original contributions presented in the study are included in the article, further inquiries can be directed to the corresponding author.

## Ethics statement

The studies involving humans were approved by the Ethics Committee of the First Affiliated Hospital of Xi'an Jiaotong University, the approval number was XJTU1AF2020LSK-273. The studies were conducted in accordance with the local legislation and institutional requirements. The participants provided their written informed consent to participate in this study. Written informed consent was obtained from the individual(s) for the publication of any potentially identifiable images or data included in this article.

## Author contributions

XYu: Data curation, Investigation, Methodology, Software, Writing—original draft. ZL: Data curation, Writing—original draft. XYa: Data curation, Writing—original draft. XW: Data curation, Writing—original draft. DN: Data curation, Writing—original draft. WL: Investigation, Supervision, Writing—review & editing. JL: Supervision, Writing—review & editing, Investigation. YM: Conceptualization, Data curation, Formal analysis, Funding acquisition, Methodology, Supervision, Writing—original draft, Writing—review & editing.
